# Jezreel

**Published:** 1918-10-05

**Authors:** Thomas Hardy


### JEZREEL (From "THE TIMES.")

### By THOMAS HARDY.

Did they catch as it were in a Vision at shut of the day—
When their cavalry smote through the ancient Esdraelon Plain,
And they crossed where the Tishbite stood forth in his enemy's way—
His gaunt, mournful Shade as he bade the king haste off amain?

On war-men at this end of time—even on Englishmen's eyes—
Who slay with their arms of new might in that long-ago place,
Flashed he who drove furiously? . . . Ah, did the phantom arise
Of that queen—of that proud Tyrian woman who painted her face?

Faint-marked they the words, "Throw her down," rise from Time eerily,
Spectre-spots of the blood of her body on some rotten wall?
And the thin note of pity that came: "A king's daughter is she,"
As they passed where she trodden was once by the chargers' foot-fall?

Could such be the hauntings of men of to-day, at the cease
Of pursuit, at the dusk-hour, ere slumber their senses could seal?
Enghosted seers, kings,—one on horseback who asks "Is it peace?" . . .
Yea, strange things and spectral may men have beheld in Jezreel!

**A poet who is a member of our staff, and who has attained encomiums and recognition for his work already published, has sent us the following tribute to the writer of this Poem.**

Thomas Hardy has never written anything more beautiful, if indeed he has ever composed so rich and austere a rhythmical music before. It is unquestionably the greatest poem occasioned by the war; the war has been worth it. It has been raised at last to the Imaginative plane; and at a stroke of the Master's pen, the Master to whom English literature already owes so much, the English arms, at least in Palestine and under Allenby, have been made one with the Host of Heaven. They are now inscribed in the book of Myth and Legend, the substantial body of history, the true Bible of the race, where the names of Hector and Achilles, Diarmuid and Maeve, Sigurd the Volsung, King Cophetua and King Ahab survive, when all the Gladstones are forgotten; and men will now think of Allenby when they read in their Bibles of Jezreel, and the English Tommy will be remembered part of the story of Jezebel.

Hardy's poem can be understood only when it is read aloud; and the three separated beats of the rhythm of each line with the cadence, or fall of the several syllables, between them, require a slow and sing-song voice; for the poem is a chant, meant, like Homer, to be chanted as a recitative to a few repeated chords upon the harp, that it may cast its spell over the listeners, and by an enchantment of their ears call up to their imaginations the Plain of Esdraelon, the figure of the prophet, and the face of the painted Tyrian looking down from her window in the wall. The rhythm of the poem slid into my ears with the softness of falling sand, and my ears themselves still retain the murmur of its music. I beg readers of The Hospital to get it by heart, if any have not already done so.—B. O.

